# Do declarative titles affect readers’ perceptions of research findings? A randomized trial

**DOI:** 10.1186/s41073-016-0018-3

**Published:** 2016-08-04

**Authors:** Elizabeth Wager, Douglas G. Altman, Iveta Simera, Tudor P. Toma

**Affiliations:** 1 Sideview, Station Road, Princes Risborough, HP27 9DE UK; 2 UK EQUATOR Centre/Centre for Statistics in Medicine, Oxford, UK; 3 University Hospital Lewisham, London, UK

**Keywords:** Research reporting, Journals, Title

## Abstract

**Background:**

Many journals prohibit the use of declarative titles that state study findings, yet a few journals encourage or even require them. We compared the effects of a declarative versus a descriptive title on readers’ perceptions about the strength of evidence in a research abstract describing a randomized trial.

**Methods:**

Study participants (medical or dental students or doctors attending lectures) read two abstracts describing studies of a fictitious treatment (Anticox) for a fictitious condition (Green’s syndrome). The first abstract (A1) described an uncontrolled, 10-patient, case series, and the second (A2) described a randomized, placebo-controlled trial involving 48 patients. All participants rated identical A1 abstracts (with a descriptive title) to provide baseline ratings and thus reduce the effects of inter-individual variability. Participants were randomized so that half rated a version of A2 with a descriptive title and half with a declarative title. For each abstract, participants indicated their agreement with the statement “Anticox is an effective treatment for pain in Green’s syndrome” using 100 mm visual analogue scales (VAS) ranging from “disagree completely” to “agree completely.” VAS scores were measured by an investigator who was unaware of group allocation.

**Results:**

One hundred forty-four participants from four centres completed the study. There was no significant difference between the declarative and the descriptive title groups’ confidence in the study conclusions as expressed on VAS scales—in fact, the mean difference between A1 and A2 was smaller for the declarative title group than that for the descriptive title group (32.6 mm, SD 27.4 vs. 39.8 mm, SD 22.6, respectively, *p* = 0.09).

**Conclusions:**

We found no evidence that the use of a declarative title affected readers’ perceptions about study conclusions. This suggests that editors’ fears that declarative titles might unduly influence readers’ judgements about study conclusions may be unfounded, at least in relation to reports of randomized trials. However, our study design had several limitations, and our findings may not be generalizable to other situations.

## Background

Many textbooks and journal instructions about how to write research articles state that the title is “the most important part of a scientific article,” and Lang has suggested that “It is the part most often read and often the only part read [1].” Despite their importance, there is no consensus on the most appropriate style for titles. Article titles may be classified as either descriptive or declarative. Descriptive (or indicative) titles describe the study topic and design. Declarative (or informative) titles state the study findings [2]. Confusingly, while many journals prohibit the use of declarative titles, a few journals encourage or even require them [3–5]. At least in some disciplines, the use of declarative titles has increased since the mid-1980s [6].

In the absence of any published evidence about their merits or dangers, opinions about title styles appear strongly held. Aronson claims “At best, declarative titles mislead; at worst they may enshrine a falsehood as a permanent truth [5].” In contrast, the instructions to authors for *Microbiology* state “A title that emphasizes the main conclusions, or poses a question, has more impact than one that just describes the nature of the study [4].”

Given these contradictory positions, and an apparent absence of evidence for such dogmas, we attempted to compare the effects of declarative and descriptive titles on readers’ perceptions about the findings of a randomized trial.

## Methods

This was a parallel group, randomized trial. Participants were doctors or senior medical or dental students attending lectures on evidence-based medicine or critical evaluation at four UK institutions (Peninsula College of Medicine and Dentistry, Plymouth and Exeter; University Hospital, Lewisham, London; Queen Mary, University of London) and the University of Split School of Medicine, Croatia. All participants were either practising medicine in the UK or taking an English language medical course and were therefore fluent in English. The study took place between January and June 2013. Since the study did not involve the collection of any personal or medical information, Research Ethics Committee approval was waived at all centres.

The study was introduced to participants as a study “about research reporting” just before or after a lecture (the timing of the study and the nature of the lecture were controlled by the local organizers). Anybody attending the lecture was eligible to take part. There was no formal enrollment, and no personal details were collected. The lecturers were instructed to emphasize that participation was voluntary and those who preferred not to take part could opt out by not taking, or not returning, a study form. The potential participants were presented with two abstracts describing studies of the efficacy of a fictitious treatment (Anticox) for a fictitious condition (Green’s syndrome). The first abstract (A1) described an uncontrolled, 10-patient, case series, and the second (A2) described a randomized, placebo-controlled trial involving 48 patients (the titles and abstracts are available in Appendix 1). The study form comprised a single sheet of A4 paper with instructions for completion at the top, followed by abstract A1 and a VAS scale on one side, and abstract A2 and another VAS scale on the other side. All participants rated identical A1 abstracts (which had a descriptive title). The A1 abstract was included to provide a baseline in an attempt to control for the fact that people’s levels of scepticism were expected to vary, and we expected some people to be “tougher” raters than others. Participants were randomized in a 1:1 ratio, so that half received a version of A2 with a descriptive title (Anticox versus placebo in Green’s syndrome: results of a randomized trial) and half received a version with a declarative title (Anticox reduces pain in Green’s syndrome: results of a randomized placebo-controlled trial).

Randomization was achieved by supplying study forms to each centre in a random sequence (achieved by the investigator (EW) shuffling the forms before posting them) and asking lecturers to distribute the forms in a way that was likely to retain the random distribution among participants (e.g., letting participants pick up forms from a mixed pile as they entered the lecture or handing out forms in a random order once participants were seated). In this way, neither the lecturer nor the participants could predict the group allocation. (The investigator, EW, was also unaware of the allocation as she was not present when the forms were distributed.) The two types of form were identical on one side (showing abstract A1) and could only be distinguished by being turned over and comparing the titles for abstract A2. Participants read and rated the abstracts without discussing them with other participants. Participants were therefore unaware of the design or purpose of the study.

For each abstract, participants indicated their agreement with the statement “Anticox is an effective treatment for pain in Green’s syndrome” using 100 mm visual analogue scales (VAS) ranging from “disagree completely” to “agree completely.” Our hypothesis was that the difference in perceptions of efficacy between A1 and A2 would be greater when A2 had a declarative title. VAS scores were measured by an investigator (EW) who was unaware of group allocation (this was achieved by masking the top of the form so that the title could not be seen while the VAS score was measured). The pre-specified primary outcome was the difference in VAS scores between A1 and A2 in the declarative and descriptive title groups. No secondary outcomes were measured.

### Statistical methods

As we could find no similar published studies, we could not predict the size of the difference nor the amount of variability within the groups that we were likely to find. It was therefore not possible to do a power calculation. We used a two-sided *t* test to analyse the difference in VAS scores between A1 and A2 in the declarative and descriptive title groups. We used analysis of covariance to take account of any differences between the groups in scores for abstract A1 [7].

An interim analysis was performed in February 2013 using data from 79 participants from three centres. This analysis was used to submit an abstract to the World Congress on Peer Review but the results were not published or communicated to the centres. The full analysis was presented in the final abstract and as a poster at the congress (September 2013).

## Results

Completed forms were returned by 144 participants (123 from UK centres, 21 from Croatia). We did not record the number of potential participants who declined to take part, but those administering the study reported that very few people opted out. Demographic data were not collected.

**Fig. 1:**
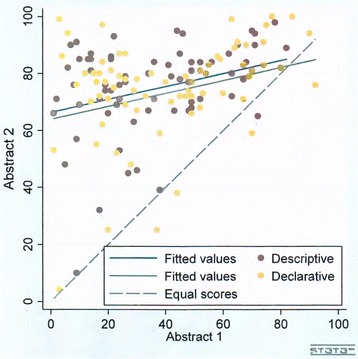
Distribution of VAS scores

There was no significant difference between the declarative and descriptive title groups’ views about study conclusions as expressed on VAS scales. In fact, contrary to our expectations, the mean difference between A1 and A2 was smaller when A2 had a declarative title (32.6 vs. 39.8 mm, *p* = 0.09, see Table 1). There was a notable difference of 6.2 mm between the groups in their mean A1 scores, however. Using the analysis of covariance to take account of that, the difference between the declarative and descriptive groups was 2.4 mm and remained non-significant, *p* = 0.42, see Table 1 and Fig. 1.

**Table 1:** Mean VAS scores for reader confidence in study conclusions for abstracts with descriptive or declarative titles (higher scores represent greater agreement with a statement on the study conclusions)

	Descriptive title group (*n* = 70) [mean (SD)]	Declarative title group (*n* = 74) [mean (SD)]	Difference between groups (95 % CI)
A1 VAS score^b^	34.4 (21.5)	40.6 (23.9)	
A2 VAS score^b^	74.2 (17.0)	73.2 (20.3)	
Difference (A2–A1)	39.8 (22.6)	32.6 (27.4)	−7.2 (–15.5 to 1.1) *p* = 0.09^a^
Adjusted difference^b^			−2.4 (–8.5 to 3.6) *p* = 0.42

^a^Two-sided *p* value from *t* test

^b^Analysis of covariance

## Discussion

We found that using a declarative title had no significant effect on readers’ perceptions of the conclusions of a randomized trial reported in an abstract. In fact, readers’ confidence in the findings was slightly lower when they received an abstract with a declarative title (but the difference was not statistically significant).

Our study had several limitations. Without an accurate estimate of the effect size or variance, it was not possible to calculate statistical power and it is therefore possible that the study was under-powered or insensitive to small effects, although we considered 144 participants to be a reasonable sample for such a study. We used abstracts rather than full journal articles so that the study could be completed quickly and be fitted in at the beginning or end of a lecture. It is possible that readers’ perceptions of abstracts and full papers, and therefore the effects of their title styles, are different, so our findings may not be generalizable to full articles. It is also possible that responses were affected by the fact that participants were attending a lecture on evidence-based medicine or critical evaluation rather than reading a publication in other circumstances. We asked participants to rate the two abstracts in an attempt to reduce inter-rater variability. However, having previously read the abstract describing the case series (A1) may have affected readers’ perceptions of the second abstract (which described a randomized trial). For example, it is possible that, having just read abstract A1, participants read abstract A2 more quickly—perhaps even skipping the title or paying less attention to it than if they had read only one publication.

We did not collect any personal details from the participants as we did not intend any sub-group analyses and did not expect sex, age, training, specialty, or experience to be important influencing factors. Our sample comprised both practising doctors and senior medical and dental students to reflect the range of people likely to read reports of clinical trials.

Despite these limitations, we believe our findings are interesting. In particular, we found no evidence to support many editors’ contention that declarative titles over-influence readers and reinforce study conclusions inappropriately.

Those who promote the use of declarative titles suggest that they may make articles more attractive (an aspect we did not attempt to measure in this study) or more effective in communicating their message. Richard Smith (when editor of *The BMJ*) wrote “journalists, who know a thing or two about getting people to read what they write, believe strongly in the use of declarative titles and the inclusion of active verbs. “Freddie Starr ate my hamster” is one of the great titles of our time. “Freddie Starr and my hamster: a personal account” would have long been forgotten [8].” The *ACP Journal Club* has suggested “By disclosing each article’s conclusion in its title, we hope to help busy clinical readers become even more efficient in their efforts to keep up with the literature [9].”

However, one motivation for doing the study, apart from contradictory journal instructions, was to test the observation from Daniel Kahneman that “A reliable way to make people believe in falsehoods is frequent repetition, because familiarity is not easily distinguished from truth” [10]. One objection to the use of declarative titles might be that they serve to repeat the study’s conclusion and, in fact, present it before readers have had a chance to assess the study. If the conclusion is unjustified, oversimplified, or misleading, this repetition could be harmful. On the other hand, if declarative titles help readers understand the article, such repetition could be beneficial. However, our hypothesis that using a declarative title would increase readers’ confidence in the study findings was not borne out.

Given the prohibition on declarative titles in numerous journals, many editors appear to side with Jeff Aaronson who denounced them in 2010 [5] and Jonah Rosner who criticised their use in the biology literature in 1990, suggesting that they were “impudent and imprudent” [11].

Despite the strongly expressed views, we found only two published studies relating to title style but none measuring effects on reader perceptions. Neville Goodman assessed study reports listed in Medline between 1970 and 1997 that included selected active verbs (such as “prevents” or “reduces”) in their title [12]. Using this method, he observed a clear increase in the use of declarative titles over that period. He also concluded that many of the declarative titles were “over-optimistic” (for example, 8 of 24 reports using the word “prevent” reported studies in which disease incidence was reduced rather than completely prevented). He concluded that “There may be arguments for reviews and editorials carrying informative titles, but [they] have little place in the reporting of research.” This may explain why declarative titles appear to be more commonly permitted (or encouraged) in journals about evidence-based medicine (such as *Evidence-Based Medicine* and *the Journal of Clinical Epidemiology*) than in journals reporting primary, clinical research.

Jonah Rosner studied the frequency of what he termed “assertive sentence titles” in biology publications from 1969 to 1989 [11]. He reported that they started being used in 1970 and had increased in frequency since then, being especially common in molecular biology. Like Goodman, he was concerned that such titles often conveyed an over-simplified interpretation of the research and he condemned their use.

More recently, Letchford and colleagues analysed 140,000 articles on the Scopus database and showed that those with shorter titles tended to attract more citations than those with longer titles [13]. They did not study the effects of title style although, interestingly, they used a declarative title for their own article.

While many journals have policies about declarative titles, less attention appears to be paid to use of the question (or interrogatory) style (such as the one we used for this article). We are aware (from our own experiences) that a few journals discourage or ban its use while others (such as *Microbiology* [3]) encourage it, but we have not studied this systematically.

## Conclusions

Our small, exploratory study neither supports nor challenges journal bans on declarative titles but may reassure editors that such titles are, perhaps, not as harmful as has sometimes been claimed. Larger studies, with a broader range of participants, in different settings and using different study designs are needed to understand this issue better. Sadly, given the cost of medical research and the importance of communicating its findings effectively and without distortion, there has been remarkably little research into the effects of reporting formats and styles on reader comprehension and perception [14, 15]. We encourage editors, publishers, and funders to remedy this and provide the basis for evidence-based research reporting.

## Appendix 1

The abstracts used in the study

A1

Effects of anticox on pain in Green’s syndrome: a case series

Background: Green’s syndrome is a rare form of food allergy characterized by pain in joints and muscles that is usually unresponsive to conventional analgesics. Anticox is a novel anti-inflammatory agent that acts on a pain pathway believed to be involved in Green’s syndrome. We tested the effect of anticox on pain in patients with Green’s syndrome.

Methods: Ten patients with suspected Green’s syndrome were given 50 mg anticox tablets, twice daily for 10 days. All patients completed a daily pain diary recording worst pain experienced that day using a 100 mm visual analogue scale (VAS). The main outcome measure was the difference in mean pain scores between Day 1 and Day 10.

Results: Eight of the 10 patients reported less pain on Day 10 than Day 1. The mean difference in pain scores was 18.3 mm (standard deviation 4.6).

Conclusions: Anticox may reduce the pain associated with Green’s syndrome.

A2 (participants were randomized to receive either the descriptive or the declarative title)

Anticox versus placebo in Green’s syndrome: results of a randomized trial

*[descriptive title]*

OR

Anticox reduces pain in Green’s syndrome: results of a randomized placebo-controlled trial

*[declarative title]*

Background: Green’s syndrome is a rare form of food allergy characterized by pain in joints and muscles that is usually unresponsive to conventional analgesics. Anticox is a novel anti-inflammatory agent that acts on a pain pathway believed to be involved in Green’s syndrome. We tested the effect of anticox on pain in patients with Green’s syndrome.

Methods: 48 adult patients with serologically confirmed Green’s syndrome were randomized and given either 50 mg anticox or matching placebo tablets, twice daily for 10 days. Participants completed a daily pain diary recording worst pain experienced that day using a 100 mm visual analogue scale (VAS). The main outcome measure was the difference in mean pain scores between Day 1 and Day 10.

Results: 44 patients completed the study and provided pain data. The mean difference in pain scores between Day 1 and Day 10 was 28.4 mm in the anticox group (n = 23) and 7.1 mm in the placebo group (n = 21). This difference was statistically significant (p = 0.01). Two patients in the anticox group reported abdominal pain.

Conclusions: Anticox reduces pain in Green’s syndrome.

## References

[1] Lang TA (2010). How to write, publish, and present in the health sciences. A guide for clinicians and laboratory researchers.

[2] Huth EJ (1999). Writing and publishing in medicine.

[3] Microbiology: instructions for authors. http://www.microbiologyresearch.org/authors/information-for-authors. Accessed 5 Aug 2015.

[4] Journal of Clinical Epidemiology. Guide for authors. https://www.elsevier.com/journals/journal-of-clinical-epidemiology/0895-4356/guide-for-authors. Accessed 14 Apr 2016.

[5] Aronson J (2010). When I use a word … Declarative titles. Q J Med.

[6] Gjersvik P, Nylenna M (2014). Declarative titles in original research papers in dermatologic journals 1974–2014. J Eur Acad Derm Venereol.

[7] Vickers AJ, Altman DG (2001). Analysing controlled trials with baseline and follow up measurements. BMJ.

[8] Smith R (2000). Informative titles in the BMJ. BMJ.

[9] Haynes RB (1994). More informative titles. ACP J Club.

[10] Kahneman D (2012). Thinking, fast and slow.

[11] Rosner JL (1990). Reflections of science as a product. Nature.

[12] Goodman NW (2000). Survey of active verbs in the titles of clinical trial reports. BMJ.

[13] Letchford A, Moat HS, Preis T (2015). The advantage of short paper titles. R Soc open sci.

[14] Wager E (2006). Publishing clinical trial results: the future beckons. PLoS Clinical Trials.

[15] Glasziou P, Altman DG, Bossuyt P, Boutron I, Clarke M, Julious S, Michie S, Moher D, Wager E (2014). Reducing waste from incomplete or unusable reports of biomedical research. Lancet.

